# Microsatellite analysis supports clonal propagation and reduced divergence of *Trypanosoma vivax* from asymptomatic to fatally infected livestock in South America compared to West Africa

**DOI:** 10.1186/1756-3305-7-210

**Published:** 2014-05-03

**Authors:** Herakles A Garcia, Adriana C Rodrigues, Carla MF Rodrigues, Zakaria Bengaly, Antonio HH Minervino, Franklin Riet-Correa, Rosangela Z Machado, Fernando Paiva, Jael S Batista, Luis Neves, Patrick B Hamilton, Marta MG Teixeira

**Affiliations:** 1 Departamento de Parasitologia, Instituto de Ciências Biomédicas, Universidade de São Paulo, São Paulo, SP, Brasil; 2 Departamento de Patología Veterinaria, Facultad de Ciencias Veterinarias, Universidad Central de Venezuela, Maracay, Aragua, Venezuela; 3 Centre International de Recherche-Développement sur l’Elevage en zone Subhumide (CIRDES), Bobo Dioulasso, Burkina Faso; 4 Instituto de Biodiversidade e Floresta, Universidade Federal do Oeste do Pará, Santarém, Pará, Brasil; 5 Hospital Veterinário, Universidade Federal de Campina Grande, Patos, Paraíba, Brasil; 6 Departamento de Patologia, Faculdade de Veterinária, Universidade Estadual Paulista Julio de Mesquita Filho, Jaboticabal, São Paulo, Brasil; 7 Departamento de Parasitologia Veterinária, Universidade Federal do Mato Grosso do Sul, Campo Grande, Mato Grosso do Sul, Brasil; 8 Departamento de Ciências Animais, Universidade Federal Rural do Semi-Árido, Mossoró, Rio Grande do Norte, Brasil; 9 Centro de Biotecnologia, Universidade Eduardo Mondlane, Maputo, Moçambique; 10 Department of Veterinary Tropical Diseases, Faculty of Veterinary Science, University of Pretoria, Pretoria, South Africa; 11 Biosciences, College of Life and Environmental Sciences, University of Exeter, Exeter, UK

**Keywords:** Nagana, Microsatellite genotyping, Clonal structure, Outbreak, Pathology, Epidemiology, South America, Africa, Animal trypanosomosis

## Abstract

**Background:**

Mechanical transmission of the major livestock pathogen *Trypanosoma vivax* by other biting flies than tsetse allows its spread from Africa to the New World. Genetic studies are restricted to a small number of isolates and based on molecular markers that evolve too slowly to resolve the relationships between American and West African populations and, thus, unable us to uncover the recent history of *T. vivax* in the New World.

**Methods:**

*T. vivax* genetic diversity, population structure and the source of outbreaks was investigated through the microsatellite multiloci (7 loci) genotype (MLGs) analysis in South America (47isolates from Brazil, Venezuela and French Guiana) and West Africa (12 isolates from The Gambia, Burkina Faso, Ghana, Benin and Nigeria). Relationships among MLGs were explored using phylogenetic, principal component and STRUCTURE analyses.

**Results:**

Although closely phylogenetically related, for the first time, genetic differences were detected between *T. vivax* isolates from South America (11 genotypes/47 isolates) and West Africa (12 genotypes/12 isolates) with no MLGs in common. Diversity was far greater across West Africa than in South America, where genotypes from Brazil (MLG1-6), Venezuela (MLG7-10) and French Guiana (MLG11) shared similar but not identical allele composition. No MLG was exclusive to asymptomatic (endemic areas) or sick (outbreaks in non-endemic areas) animals, but only MLGs1, 2 and 3 were responsible for severe haematological and neurological disorders.

**Conclusions:**

Our results revealed closely related genotypes of *T. vivax* in Brazil and Venezuela*,* regardless of endemicity and clinical conditions of the infected livestock. The MLGs analysis from *T. vivax* across SA and WA support clonal propagation, and is consistent with the hypothesis that the SA populations examined here derived from common ancestors recently introduced from West Africa. The molecular markers defined here are valuable to assess the genetic diversity, to track the source and dispersion of outbreaks, and to explore the epidemiological and pathological significance of *T. vivax* genotypes.

## Background

Animal trypanosomosis (Nagana) caused by *Trypanosoma vivax* can be a highly debilitating disease in African and South American livestock [1,2]. In Africa, *T. vivax* is highly prevalent in both tsetse-infested and tsetse-free regions. It is considered an important pathogen in Burkina Faso [3,4], Ghana, Zambia [5], Nigeria [6], Uganda [7], Ethiopia [8,9], Sudan [10] and Cameroon [11]. The cyclical transmission of *T. vivax* is limited to tsetse flies; mechanical transmission by other biting flies allows *T. vivax* to spread in some tsetse-free African regions and to Central and South America, where it is disseminated by tabanids and stomoxes [1,2]. In South America, the transplacental transmission of *T. vivax* also plays an important role in its epidemiology [12,13].

In Africa, bovids and suids are hosts of *T. vivax*, and this species can be pathogenic in equines, camels, cattle, goats and sheep, whereas wild ungulates serve as reservoirs [14-19]. In South America, wild reservoirs are unknown and *T. vivax* can be pathogenic to cattle, sheep, goats and horses [12,20-23]. The main manifestation of acute *T. vivax* disease is devastating anaemia and compromised cardiac function [14,15,22,24,25], followed by the invasion of the central nervous system [20,23,26,27], abortion, stillbirth, and testicular and ovarian damage [12,13,28]. Disease severity and particular clinical signs have been associated with geography, prior infections, health conditions, and livestock species and breeds. In general, West African (WA) isolates are more pathogenic to livestock than East African (EA) *T. vivax* isolates*,* but wasting disease with haemorrhagic syndromes has been reported in Kenya and Uganda [14,15,24]. Outbreaks of acute haematological and neurological disorders with high mortality have been reported affecting cattle, goats, sheep and horses throughout non-endemic Brazilian regions [20-22,29]. In South American (SA) regions of enzootic stability (Amazonian lowlands, Venezuelan Llanos and Brazilian wetland of the Pantanal), infections are mostly asymptomatic in cattle, buffaloes and sheep, all showing low parasitaemias [1,30-33].

*T. vivax* is endemic in many countries in Central and South America. The first reports of *T. vivax* in the New World were in French Guiana (1919), Venezuela (1920), the Caribbean Guadalupe and Martinique (1926 and 1929), and Colombia (1931), which are former French and Spanish colonies. In Brazil, a former Portuguese colony, *T. vivax* was first recorded in cattle (1946) and buffaloes (1972) in Amazonia [1,2]. Based on historical livestock introduction and limited parasite genetic evidence, it has been suggested that *T. vivax* was introduced into the Americas via West African cattle [1,2,30,34-37]. Cattle, horses, sheep, goats, donkeys and pigs were first brought to the Americas on the voyage of Columbus in 1493, and for centuries the transport of Iberian and African livestock to the Americas followed the routes of the African slave trade [38-40]. Therefore, cows, goats, sheep and equines brought by the colonisers could be responsible for the introduction of *T. vivax* into the Americas at different times and places.

Early studies comparing African and American *T. vivax* isolates using molecular markers showed a relevant similarity between SA (Colombian) and WA isolates [36,37]. The close relationship between SA and WA *T. vivax* was corroborated by phylogenetic analyses of cattle isolates from Brazil (3 isolates), Venezuela (one isolate), West Africa (Y486 from Nigeria) and East Africa (IL3905 from Kenya) using Spliced Leader [30], SSU and ITS rDNA [34], and Cathepsin L-like [35] sequences. Previous studies evidenced high divergence separating SA/WA from EA parasites, and also revealed substantial divergence among EA isolates from Kenya and Mozambique [30,34,35]. Highly divergent isolates from Tanzania (EA) were reported from tsetse flies by comparing gGAPDH sequences [41,42], and also in wild animals through ITS rDNA analysis [19]. Cattle isolates from Ghana clustered with SA/WA isolates, while Zambian isolates were more related to Kenyan *T. vivax*[5]. Genetic studies have revealed more relevant genetic diversity in EA *T. vivax* compared to populations in WA [17,19,30,34,35,41-43]. Unfortunately, the use of different molecular markers prevented a global comparison of data from all previous studies.

The genetic studies on *T. vivax* were mostly based on molecular markers that evolve too slowly to resolve the relationships between SA and WA populations and, hence, were unable to uncover the recent history of this parasite in the New World. Microsatellite multiloci genotype (MLG) analysis can reveal cryptic genetic diversity, population structure and the origin of parasites, as have been shown for *T. brucei* spp. [44-47] and *T. congolense*[48,49]. The most comprehensive genetic study of *T. vivax* by MLG analysis was restricted to isolates from donkeys in The Gambia and results suggested a clonal population [50]. More considerable MLG polymorphisms were demonstrated in Cameroon [51] and Uganda [52], despite the few isolates examined.

In this study, we analysed polymorphisms in 7 MST loci in isolates from across South America (39 from Brazil, 7 from Venezuela and one from French Guiana) and West Africa (12 isolates from The Gambia, Burkina Faso, Ghana, Benin and Nigeria) aiming to assess the genetic repertoire and phylogenetic relationships at continental and intercontinental levels and, hence, to understand the introduction and dispersion of *T. vivax* in South America. The comparison of isolates from asymptomatic livestock living in areas of enzootic stability and isolates from sick animals from outbreaks, exhibiting a range of haematological and neurological signs and several fatal cases, as well as repeat sampling from the same areas, allowed us to examine potential links between genotype and disease, outbreaks, host species and virulence in the context of spatial-temporal changes.

## Methods

### *T. vivax* samples from endemic settings and outbreaks

**Figure 1:**
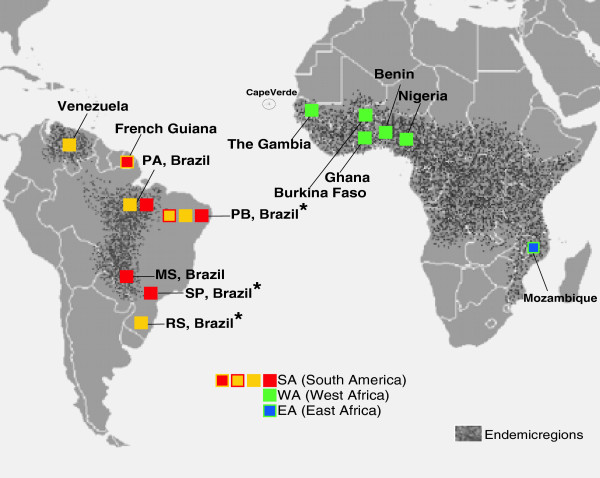
**Geographical origin of *Trypanosoma vivax *isolates from South America and Africa and microsatellite genotype (MLGs) distribution.** Map showing the different localities of *T. vivax* isolates used in this study, including samples from endemic regions and from outbreaks (_*_) in non-endemic areas. Brazilian States: PA, Para; PB, Paraiba; MS, Mato Grosso do Sul; SP, São Paulo; RS, Rio Grande do Sul. Coloured symbols (boxes) correspond to individual MLG or clusters of MLGs defined with the analysis of 7 microsatellite loci (Figures 2 and 3).

The identification of *T. vivax* in blood samples was performed using a *T. vivax*-specific PCR assay (TviCATL-PCR) based on Cathepsin-L gene [35]. The South American isolates were from blood samples collected at widely distributed locations in Brazil and Venezuela (Figure 1) from cows, buffaloes, sheep and horses. In addition, one sheep isolate from French Guiana was included (Table 1). All SA isolates, including their host species and geographic origin, and clinical signs of infected livestock are detailed in an additional table [see Additional file 1].

**Table 1:** **Geographical and host origin, clinical manifestation and microsatellite loci genotyping data from *Trypanosoma vivax *** **isolates included in this study**

**Geographical origin**	**Host species (N**^**o **^**of isolates)**	**Clinical manifestation**	**MLG**	**Genotypic diversity (N**^**o **^**of MLGs/isolates)**	**Allelic composition (N**^**o **^**of different alleles)**
**South America (SA)**					
**Brazil**					
Endemic area	Buffalo (17)	Asymptomatic^a^	1,3,6		
	Cow (4)		1, 2		
Non-endemic area	Buffalo (1)	Asymptomatic^a^	5		
	Cow (1)	asymptomatic^a^	5		
	Sheep (2)	Asymptomatic^a^	4,5		
(outbreak)	Cow (2)	Asymptomatic^a^	1		
(outbreak)	Sheep (11)	Severe/lethal^b^	2		
(outbreak)	Horse (1)	Severe/lethal^b^	3		
*Total*	(39)			0.15 (6/39)	16
**Venezuela**					
Endemic area	Cow (3)	Asymptomatic^c^	7,9,10		
	Buffalo (2)	Asymptomatic^c^	8,10		
	Sheep (2)	Asymptomatic^c^	8		
*Total*	(7)			0.57 (4/7)	15
**French Guiana**					
Endemic area	Sheep (1)	Symptomatic^c^	11	(1/1)	12
*Total SA*	(47)			0.23 (11/47)	21
**West Africa (WA)**					
Burkina Faso	Cow (4)	Symptomatic^c^	12,13,14,15	(4/4)	
Ghana	Cow (4)	Symptomatic^c^	16,17,18,19	(4/4)	
Benin	Cow (2)	Symptomatic^c^	20,21	(2/2)	
Nigeria	Cow (1)	Symptomatic^c^	22	(1/1)	
The Gambia	Cow (1)	Symptomatic^c^	23	(1/1)	
*total WA*	(12)			1.0 (12/12)	41
**East Africa (EA)**					
Mozambique	Cow (1)	Symptomatic^c^	24	(1/1)	
	Nyala (1)	ND^d^	25	(1/1)	
*Total EA*	(2)			1.0 (2/2)	17
*Total SA + WA + EA*	61			0.41 (25/61)	

a, normal PCV and very low parasitemia; b, high parasitemia severe anemia (low PCV values) and nervous signs; c, low PCV values and low parasitemia; d, high parasitemia. Details of each isolate are in Additional files 1 and 2.

A total of 47 South American isolates were genotyped by MST analyses: 39 from Brazil, 7 from Venezuela, and one from French Guiana. Furthermore, 12 samples from West Africa and two from East Africa were also analysed. *T. vivax* isolates from animals infected during different Brazilian outbreaks (Figure 1) were compared. Isolates from very sick sheep were from an outbreak in the northeast (state of Paraiba) that affected a flock and caused severe haematological and neurological alterations along with several deaths [20]; other outbreaks in cows and sheep occurred previously in the same region [29]. Two isolates were from a cow and a horse that showed haematological and neurological disturbances in sequential outbreaks in the southern region (state of Rio Grande do Sul) [21]. Two isolates were from an outbreak in the south-eastern region (São Paulo) from cattle that exhibited severe haematological changes [22]. The Venezuelan isolates were from cattle, buffalo and sheep from the endemic Llanos region (Figure 1), where the infected animals are generally asymptomatic but can sporadically show moderate parasitaemia and anaemia [32,33]. The isolates from West Africa included in this study were all from low parasitemic cattle showing mild anemia and were collected in *T. vivax* endemic settings in Burkina Faso, The Gambia, Ghana, Benin and Nigeria. Isolates from Mozambique were obtained from cattle and a nyala antelope [17]. The field-collected samples were designated as primary samples, and the laboratory samples represent isolates expanded in experimental animals (Table 1). *T. vivax* from Africa, including host and geographic origin, and clinical signs of infected animals are detailed in an additional table [see Additional file 2].

### Ethical approval

The handling of livestock was performed in strict accordance with good animal practice as defined by the World Organization for Animal Health guidelines and approved by Veterinary Scientific Boards of the Centre International de Recherche Développement sur l’Elevage en zone Sub-humide in Burkina Faso, Universidade Eduardo Mondlane in Mozambique, and Brazilian Universities that participated in this study. The whole project was conducted in strict accordance with the recommendations of the Brazilian National Council of Animal Experimentation (http://www.cobea.org.br/) and approved by the Animal Experimentation Ethics Committee from the Institute of Biomedical Center, University of São Paulo, Brazil (CEP-ICB nº 317/09).

### Microsatellite markers and analyses

Data from the *T. vivax* Y486 Genome at the Sanger Institute (http://www.sanger.ac.uk/Projects/T_vivax/) were used for searching MST through the Microsatellite Repeats Finder (http://tandem.bu.edu/trf/trf.html) program. MST loci were selected from 5 different scaffolds reducing physical linkages. We designed primer pairs for 14 loci, and one primer from each locus was labelled with FAM. An additional table shows the sequences of all primers, genes and genome locations, and MST motifs of all loci examined [see Additional file 3].

The 14 primer pairs were initially tested using purified DNA from the reference Y486 *T. vivax*, two further isolates from Brazil and Mozambique, and other species (*T. b. brucei*, *T. evansi* and *T. congolense*). Seven of the 14 primer pairs (MST loci 4, 7, 8, 10, 11, 13 and 15) were specific for *T. vivax*. The PCR amplifications were performed in a 25 μl reaction mixture consisting of ~20 ng of DNA, 100 pmol of each primer, 200 mM of each dNTP, 10 mM Tris–HCl (pH 8.3), 3.0 mM MgCL_2_, 7.5% (v/v) dimethyl sulphoxide, 0.1 mg/ml bovine serum albumin and 1.0 U Taq DNA polymerase. The amplification conditions were as follows: initial denaturation at 95°C for 3 min followed by 30 cycles of 30 s at 95°C, 30 s at the specific annealing temperature for each marker, 1 min at 72°C, and a final extension at 72°C for 5 min. The annealing temperatures were: 55°C (MST 4, 8 and 11); 58°C (MST 13 and 15); or 60°C (MST 7 and 10). The allele sizes for each locus were determined using a capillary-based sequencer and the Gene Mapper® software with Gene Scan 500-ROX size standards (Applied Biosystems). The individual peaks defined each allele, and the data set from the 7 loci defined each MLG.

Allele frequencies and estimates of genetic variation within populations (average numbers of alleles per locus, allelic richness and the means of the expected, total expected and observed heterozygosity) were calculated using ARLEQUIN 3.5 [53]. Genotypic diversity was estimated as the number of different MLGs divided by the total number of isolates. Conformation to Hardy-Weinberg equilibrium, as a test of the non-random associations of alleles within diploid individuals, and the linkage disequilibrium between all pairs of loci, as a test of the non-random association of alleles at different loci, were also determined in ARLEQUIN 3.5 [53]. The Fixation Index (F_IT_), as a measure of an overall inbreeding coefficient, was determined in GenAlEx 6 [54]. F_IT_ values range from -1 to 1, where values close to zero are expected under random mating, substantial positive values indicate inbreeding, and negative values indicate an excess of heterozygosity.

To examine the relationships between SA and WA *T. vivax*, the pairwise measure of shared allele distances from the microsatellite dataset was calculated using the program POPULATIONS v1.2.30 beta [55], and dendrograms based on MLGs were constructed using the DAS, shared allele distance [56] and the neighbour-joining (NJ) method (bootstrap based on 100 replicates). Principal component analyses (PCA) of the MLGs were performed in GenAlEx 6 [54]. A Bayesian clustering approach as implemented in the program STRUCTURE v2.3.3 [57] was employed to estimate the number of genetically differentiated clusters (*K*) within the data set. Log-likelihood values for each value of *K* (ranging from 1 to 16) were evaluated from all MLGs by running the STRUCTURE program with 300,000 repetitions for three replicates (burn-in = 100,000 iterations), and the most likely value of *K* was assessed by the method of Evanno *et al*. (2005) [58]. STRUCTURE analysis was performed as described previously for *T. brucei* ssp. MLG studies [45-47].

## Results

### Microsatellite multilocus genotyping and relationships of *T. vivax* in South America and West Africa

**Figure 2:**
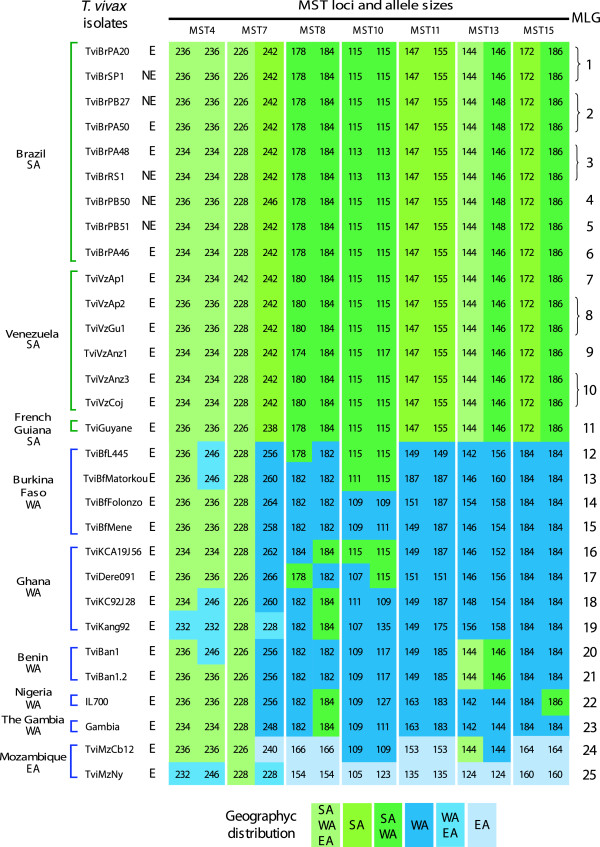
**Microsatellite genotypes (MLGs) of *Trypanosoma vivax*.** The *T. vivax* isolates included in this figure are representatives of all MLGs found in isolates from South American (SA) endemic (E) and non-endemic (NE) areas (16 SA isolates); plus 12 isolates from West Africa (WA) and two from East Africa (EA) (see details of isolates in Additional files 1 and 2). Samples were genotyped at 7 microsatellite loci (MST-4, 7, 8, 10, 11, 13, 15) using primers specific to *T. vivax*. Colours represent the alleles shared by isolates from different geographical regions.

The MLG analysis of *T. vivax* populations included 47 SA and 12 WA isolates, most genotyped directly from blood samples, thereby avoiding parasite selection by inoculation in experimental animals. Laboratory isolates submitted to successive passages in animals were included for comparison (Table 1, Additional file 1). The total number of MLGs found was 25, consisting of 11 MLGs in 47 SA samples (MLGs 1–11) and 12 MLGs in 12 WA samples (MLGs 12–23). Therefore, WA *T. vivax* displayed considerably greater genetic diversity than the SA parasites. Among the SA isolates, MST11 and MST15 were invariant, the MLGs generally differed at only one MST locus, and many samples shared the same MLG (Figure 2). An additional file shows results for all SA isolates [see Additional file 1]. The WA MLGs were defined by unique alleles found in Burkina Faso (MLGs 12–15), Ghana (16–19), Benin (20 and 21), Nigeria (22) and The Gambia (23). Although no MLG was shared between SA and WA *T. vivax*, most alleles were shared by the two populations. The SA isolates showed high homogeneity, with small variability restricted to four loci. While 12 alleles were shared between WA and SA*,* only 4 alleles were shared between EA and SA *T. vivax*. The two EA isolates included in the analyses were assigned to two unique MLGs (24 and 25) clearly separated from both SA and WA MLGs (Table 1; Figure 2).

To illustrate the MLG repertoire and allelic composition from SA isolates, 16 samples representing all allele profiles (MLGs) identified across the host species and geographical range were compared with the African isolates (Figure 2). An additional file shows the data from the remaining SA isolates [see Additional file 1]. Eleven MLGs (1–11) were identified in SA and the closely related MLGs 1–6 were detected exclusively in Brazil. MLGs 1–2 were the most prevalent (~79.5%), probably due to many samples from Amazonia and Semiarid. MLG 1 was found in 70% of the Amazonian asymptomatic buffaloes, which also harboured MLGs 3 and 6, whereas MLG 2 was detected in asymptomatic cattle in this region. No MLG was exclusive of sick animals. MLG 1 was found in fatally infected cows from an outbreak in the south-eastern region [22]. MLG 2 was detected in a sheep outbreak of fatal infection with nervous signs in north-eastern Brazil [20] where, one year later, MLG 5 was detected in asymptomatic sheep, cattle and buffalo, and MLG 4 in asymptomatic sheep. MLG 3 was found in a horse with fatal infection in the southern region where cattle were reported infected with *T. vivax* three years before [21].

**Figure 3:**
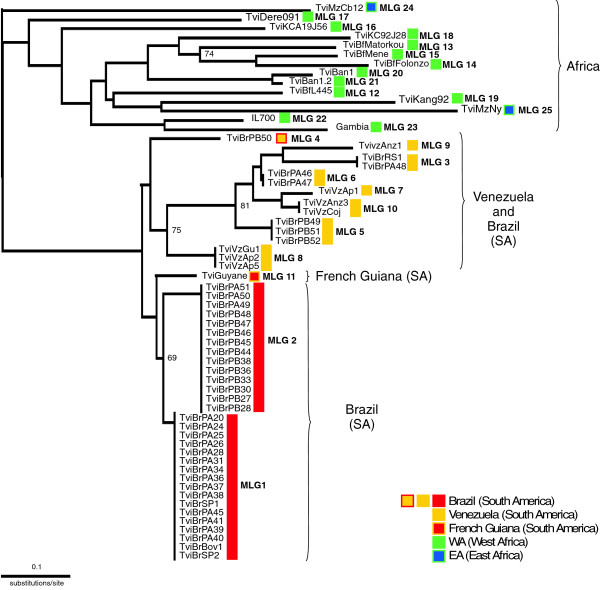
**Phylogenetic relationships of *Trypanosoma vivax *from South America and Africa based on microsatellite genotypes (MLGs).** Neighbour-joining dendrogram based on shared allele distance; bootstrap values are shown for the major nodes. Symbols represent the isolates and respective MLGs. Colours represent the following groups: **a)** formed exclusively by homogeneous Brazilian isolates (red) and one isolate from French Guiana (red with yellow borders), **b)** moderately heterogeneous Venezuelan (yellow) and Brazilian (yellow/red) isolates, **c)** highly heterogeneous isolates from West (green) and East (blue/green) Africa. Codes of the isolates in South American (TviBr = Brazil, TviVz = Venezuela, TviGuyane = French Guiana), West African (TviBf = Burkina Faso, TviK/TviDere = Ghana, TviBan = Benin, IL700 = Nigeria, Gambia = The Gambia) and East African (TviMz = Mozambique).

**Figure 4:**
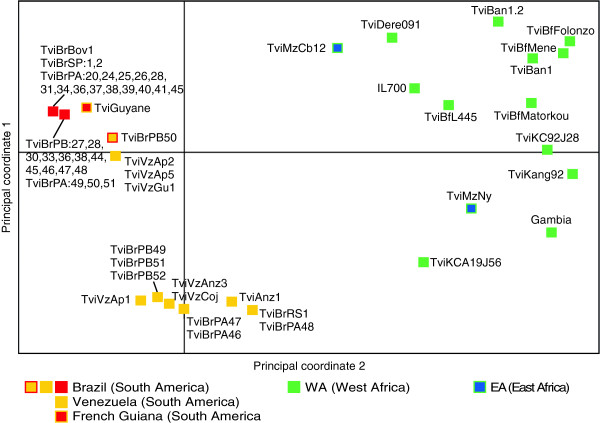
**Principal component analysis (PCA) of *Trypanosoma vivax *populations.** PCA analysis of 61 isolates from South America (47), West Africa (12) and East Africa (2) based on data from 7 microsatellite multiloci genotyping (MLG). Multidimensional scaling plots of genetic distances: Principal coordinate 1 separates South American and African populations besides evidencing the genetic diversity within both populations, notable in Africa. Principal coordinate 2 separates Venezuelan from most isolates from Brazil highlighting the more homogeneous Brazilian population compared to populations in Venezuela. Codes represent individual isolates and colours their respective genotypes (MLGs) and groups of isolates from South America (TviBr = Brazil, TviVz = Venezuela, TviGuyane = French Guiana), West Africa (TviBf = Burkina Faso, TviK = Ghana, TviBan = Benin, IL700 = Nigeria, Gambia = The Gambia) and East Africa (TviMz = Mozambique).

**Figure 5:**
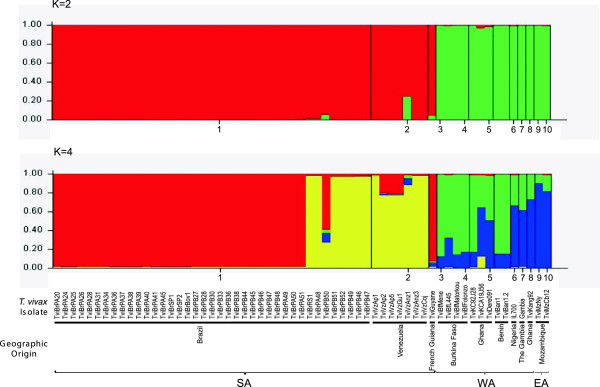
**Genetic analysis of *Trypanosoma vivax *isolates from South America and Africa.** Bayesian clustering (STRUCTURE analysis plots) of 61 isolates based on 7 microsatellite loci are shown for K = 2 (two main clusters corresponding to South American and African populations) and K = 4 (partition of South American samples in two main clusters, one formed exclusively by Brazilian isolates and the other comprising all Venezuelan and some Brazilian isolates. Each cluster of isolates is represented by a different colour according to geographic region; thin black horizontal lines separate the populations. Codes of isolates in South America (TviBr = Brazil, TviVz = Venezuela, TviGuyane = French Guiana), West Africa (TviBf = Burkina Faso, TviK = Ghana, TviBan = Benin, IL700 = Nigeria, Gambia = The Gambia) and East Africa (TviMz = Mozambique).

To represent the relationships between *T. vivax* isolates, we constructed a NJ dendrogram based on pairwise distances between the MLGs (Figure 3). This analysis showed the separation between the WA and SA isolates. In addition, most Brazilian isolates sharing highly similar MLGs clustered together, whereas the Venezuelan isolates clustered closely with some of the Brazilian isolates, forming a heterogeneous cluster of isolates assigned to different MLGs. The WA isolates formed a highly heterogeneous cluster; only two samples from Benin and three out of the four samples from Burkina Faso grouped together. The dendrogram was consistent with the clustering analyses (Figures 4 and 5), even though most nodes had low bootstrap support, most likely due to the low number of isolates and loci examined.

We further assessed the relative genetic differentiation between the clusters using PCA (Figure 4), which also allowed for the visualisation of the three main clusters evidenced by the inferred dendrogram. PCA revealed a cluster of isolates exclusively from Brazil, with other clusters formed by isolates from Venezuela and some isolates from Brazil; clustering in different quadrants indicating sub-structuring within SA populations. The last cluster consisted exclusively of WA isolates distributed in two quadrants (Figure 4). Using the method of Evanno *et al.*[58], the inferred most-likely number of genetically distinct clusters (K) in the STRUCTURE analysis was K = 2, corresponding to very well separated SA and WA *T. vivax* populations. However, additional groups were present in K ≥ 3, with the split of all the Venezuelan and some Brazilian isolates (Figure 5), in agreement with both the NJ dendrogram and PCA analyses (Figures 3 and 4).

### Population structure of *T. vivax* in South America

The population structure of *T. vivax* in South America was examined by analysing genotypic diversity among isolates, multilocus standardised index of association, and inter-population differentiation. All 7 MST markers were polymorphic in South America; because none of the samples displayed more than two alleles per single locus, all samples are diploid and have a single genotype. Loci MST-7, MST-8, MST-10 and MST-13 displayed between 3 and 5 alleles per locus, whereas MST-4, MST-11 and MST-15 showed two alleles (Figure 2). The allelic composition of the SA isolates was 21 for 47 isolates examined with 9 unique alleles. Nei’s unbiased genetic diversity (H_s_) ranged from 0.1 to 0.6 (average of 0.5) assuming neutrality. The results indicate a low genetic diversity of *T. vivax* in South America compared to West (41 alleles for 12 isolates with 9 unique alleles) and East Africa (17 alleles for only two isolates with 10 unique alleles) (Table 1).

The analysis of non-random association of alleles revealed a significant deviation (P < 0.05) from Hardy-Weinberg equilibrium predictions at all loci, and this observation was linked to the global heterozygote excess, with most loci at, or close to, heterozygote fixation. As expected for heterozygote excess, the F_IT_ values for these loci ranged from -0.57 to -1.0. Two loci (MST-4 and MST-10) showed heterozygote deficits, with F_IT_ values of 1.0 and 0.79, respectively. However, the mean observed heterozygosity for SA isolates was higher than expected (0.71 vs. 0.47), and the mean F_IT_ value reflects this result (-0.31). A significant linkage disequilibrium (P < 0.05) was observed in the majority of loci combinations.

The results support a clonal population structure of *T. vivax* in South America. The limited number of isolates prevented statistical analysis of the African populations. Our attempts to include several EA samples in the MLG analysis were unsuccessful, most likely due to the large genetic divergence within EA genotypes precluding the use of primers based on the WA *T. vivax* Y486 genome.

### Cryptic diversity within South American *T. vivax* infecting livestock in endemic areas and outbreaks

The MLG analyses of SA *T. vivax* carried out in this work did not support association with host species or clinical manifestation: buffaloes harbour MLGs 1, 3, 5 and 6; cattle harbour MLGs 1, 2 and 5; and sheep harbour MLGs 2, 4 and 5 (Table 1). An additional file shows the MLGs designed for all SA isolates characterized in this study [see Additional file 1].

MLGs 7–10 were exclusive to Venezuelan samples from animals in endemic regions: MLG 8 and MLG 10 were found in buffaloes, MLG 7, 9 and 10 in cattle, and MLG 8 in sheep. The genotype MLG 8 was shared between buffalo and sheep from neighbouring regions (Apure and Guárico), where animal interchange is intense, whereas more divergent genotypes were found in cattle from a more distant region (Anzoátegui) (Figure 2). The results suggested that some spatial sub-structuring separated Brazilian and Venezuelan isolates. Nevertheless, some samples from Brazil were more closely related to those from Venezuela than to other Brazilian samples in the NJ dendrogram, PCA and STRUCTURE analyses. The only isolate from French Guiana included in this work was assigned to an exclusive genotype (MLG 11) (Table 1; Figures 2 and 3).

## Discussion

This study of the population genetic structure of *T. vivax* from South America and West Africa through MLG analysis corroborated the close phylogenetic relationships among SA and WA genotypes previously suggested based on a much smaller sample size and limited geographical sampling based on different markers such as kDNA [37], Spliced Leader [30], SSU and ITS rDNA [19,34], Cathepsin L-like [5,35] and gGAPDH [41,42] sequences. The results from MLG analysis of SA and WA isolates characterized in this work provides additional support to previous hypotheses that have emerged over the last decades that *T. vivax* was introduced from West Africa into South America [30,34-37,42]. However, no MLG was shared between SA and WA *T. vivax,* revealing that despite closely related SA and WA *T. vivax* are genetically distinct. This finding may indicate that mutation events occurred in either WA or SA, reflecting disconnected populations, or it may simply reflect the absence in this study of the African genotypes that represent the source of *T. vivax* in South America. In addition, the MLG analysis revealed small but significant genetic differences within SA populations with evidence of some sub-structuring: most Brazilian isolates grouped in a single cluster, with a second cluster containing all Venezuelan isolates along with a few Brazilian isolates from different regions.

Additionally, our findings suggest, for the first time, that genotypes from common ancestry give rise to closely related but genetically different and widespread populations in South America. However, despite our analysis including samples from former Portuguese, Spanish and French colonies in Africa, the most probable origin of *T. vivax* brought to South America, we have no evidence about the route or the livestock species that carried this trypanosome to the Americas. The shipment of livestock to the New World begin ~500 years ago, with intermediate ports in the Atlantic Islands of Cape Verde and Canarias [38-40] allowing the mixture of animals from Africa and Europe, where there are no past or present day reports of *T. vivax.* Therefore, the exact geographic origin in West Africa, how many times and locations of introduction, and the livestock species that carried *T. vivax* to the New World all require further broader analysis across Africa and the Americas.

The population structure observed in the present study supports the hypothesis of clonal propagation of *T. vivax* in South America. Evidence is provided by significant levels of linkage disequilibrium between most MST loci, the absence of recombinant genotypes and an excess of heterozygosity. A clonal structure was also suggested by a previous MLG analysis from The Gambia, where *T. vivax* was shown to be clonally propagated among donkeys [50]. It would be interesting to evaluate population structures from other regions of Africa, particularly in East African localities where tsetse flies are abundant and greater levels of *T. vivax* diversity occur [17,19,34,41,42,59], as well as in endemic tsetse-free areas of Sudan and Ethiopia [8-10,59].

According to the NJ dendrogram, PCA and STRUCTURE analyses, the *T. vivax* isolates within the main clusters were more similar in geographical origin than in date of sampling, species of origin, or clinical manifestations. The exception was the two EA (Mozambique) isolates that clustered together with those from WA. These isolates also clustered with those from WA and SA using the conserved gGAPDH sequences [41,42]. The phylogenetic analysis based on Proline-Racemase (TviPRAC) gene sequences showed the two EA isolates included in this study clustering closer to SA and WA genotypes than to other EA isolates (Caballero *et al*. in preparation). Further evidence that some EA isolates are closely related to WA/SA genotypes came from a recent study of *T. vivax* in Ethiopia showing isolates from tsetse-free areas sharing conserved TviPRAC sequences with WA/SA isolates, whereas isolates from tsetse-infested regions showed divergent sequences [59]. Therefore, increasing evidences are supporting the existence in EA of *T. vivax* genotypes ranging from very closely related to highly distant to WA/SA populations. In addition, results based on polymorphic ITS rDNA sequences were sufficiently polymorphic, in agreement with unique allelic composition, to clearly distinguish the two EA samples included in this study [17,19,34].

There is no evidence that the highly divergent *T. vivax* that has been described from East Africa was introduced into the South America. The greater genetic diversity of *T. vivax* in East Africa compared to West Africa may be related to natural transmission cycles involving a range of wild ungulate, and tsetse species in natural reserves of wildlife [17,19,35,41,42]. Further analyses are required to evaluate whether comparable diversity also occurs in EA livestock and to evaluate whether diversity in West Africa has been underestimated because all isolates examined were from livestock. Studies across African countries are of fundamental importance to better understand the events shaping the genetic repertoire of *T. vivax* over a long evolutionary history in Africa*.* Similarly, more comprehensive analyses are required to explore the genetic diversity of *T. vivax* across South and Central America to better hypothesize about the history of *T. vivax* in the New World.

Brazilian and Venezuelan populations shared a much more similar allelic composition compared to those found in West Africa, providing evidence that the *T. vivax* population we have investigated in South America originated from WA genotypes of a common ancestry. Recent common ancestors is also suggested by the fact that all SA MLGs we have identified can be explained by mutations that, in general, produced only two repeat MST units. This hypothesis is supported by the close relationships among *T. vivax* from the Llanos of eastern Venezuela to a vast geographical range including northern, central, north-eastern, south-eastern, and southern Brazilian regions. Given the significant genetic diversity found in WA compared to SA countries, the introduction of divergent genotypes would most likely have resulted in greater genetic diversity in SA *T. vivax* populations, unless there was strong selection for particular genotypes post-importation. The more homogeneous SA population of *T. vivax* could result from a population bottleneck effect when the parasite adapted to exclusively mechanical transmission.

The comparison of *T. vivax* from asymptomatically to fatally infected livestock from endemic settings and outbreaks, respectively, revealed that of the 6 MLGs detected in Brazil, only three (MLGs 1–3 sharing highly similar allelic composition) were responsible for the outbreaks. This result could suggest that outbreaks can occur due to clonal expansions underlying more virulent populations. However, these three MLGs were also found in asymptomatic animals from endemic areas. The sharing of MLG between endemic and outbreak sites supports the hypothesis that the parasite sources of the outbreaks were asymptomatic animals from endemic settings introduced into *T. vivax-*free regions causing severe acute disease, regardless of the genotypes introduced, into the naïve hosts. Although our study found no strong association between disease and genotype, the analyses of additional loci and samples may reveal genotypes varying in virulence and pathogenicity. In fact, sheep and calves experimentally infected with a MLG1 Brazilian isolate from the Pantanal region exhibited very low parasitemia and lacked signs of pathogenicity [31]. In contrast, MLG2 isolates from cattle and sheep from outbreaks in the semiarid region have been shown to be highly virulent for sheep and goats [12,13,27,28].

It will be useful to assess the temporal stability of *T. vivax* genotypes in longitudinal surveys in specific hosts and geographic areas. Our preliminary data did not indicate substantial temporal changes of genetic profiles of parasites in livestock living in areas of enzootic stability. Indeed, the highly prevalent MLG1 genotype detected in Amazonian buffaloes in 2008 and 2009 was also found ten years earlier in cattle from the Pantanal, which is also an endemic area, and in cattle from an outbreak of acute disease with nervous compromises in the south-eastern region that occurred in 2008 [29,31]*.* In contrast, in a farm in non-endemic north-eastern Brazil, where MLG2 was isolated from an outbreak of high mortality in sheep in 2008, only MLGs 4 and 5 (genotypes never found in endemic areas) were found in asymptomatic sheep, buffalo and cattle one year after the outbreak, when only the sick animals were submitted to treatment [20].

Despite our efforts to obtain a representative number of isolates, limitations of this study were the very low parasitemia and the small number of isolates and loci examined. In addition, besides the low number of WA samples, they were limited to cattle and results could be biased towards isolates selected by these animals. Host selection has been considered as an important determinant of the population structure of *T. brucei*[60]. Nevertheless, this is the first MLG analysis comparing SA populations, and the results are the first step towards the understanding of population structure and genotype repertoire of *T. vivax* throughout distinct epidemiological scenarios. However, more substantial sampling from specific host species and regions are needed to avoid complications of sub-structuring by host, time or space. The recent definition of unique repertoires of *VSG*[61], trans-sialidase enzymes involved in parasite virulence [25], and genes differentially expressed between *T. vivax* from Venezuela and *T. vivax* Y486 from West Africa [62] have all provided new opportunities to select new markers that are useful for comparing the phenotypic and genotypic diversity of *T. vivax*.

## Conclusions

Knowledge of the genetic structure of populations is critical to investigate the origin, dispersion and impact of genetic variation on pathogenicity and epidemiology of *T. vivax in South America*. The MLGs analysis from *T. vivax* across Brazil and Venezuela support both clonal propagation and the hypothesis that the isolates examined here derived from highly closely related ancestors recently introduced from West Africa into the Americas. Genetic repertoire was reduced in South America compared to West Africa. Here, we conducted the first molecular comparison of *T. vivax* from asymptomatically to fatally infected livestock that exhibited a range of haematological and neurological disorders. Our findings found no strong association between genotype, host species, virulence and pathogenicity.

The increasing number of Brazilian *T. vivax* outbreaks resulting in high mortality of cows, sheep, goats and horses, highlights the importance of adopting approaches to monitor the spread of *T. vivax* and the possible selection and emergence of genotypes. The molecular markers employed in this study are valuable for assessing the genetic diversity of American and African populations, for reconstructing the pathways of *T. vivax* introduction and dispersion into the Americas, and for determining whether particular genotypes emerged locally or were imported allowing tracking the source of parasites in outbreaks.

## Competing interests

The authors declare that they have no competing interests.

## Authors’ contributions

MMGT, HAG, ACR, PBH conceived this study, analysed and interpreted the data set and prepared the manuscript. HAG, CMFR, ZB, AHM, FRC, RZM, JSB, FP and LN participated in field studies, collecting blood samples, examining the infected animals and described all clinical and epidemiological scenarios. HAG, ACR, CMFR, PBH carried out the molecular analyses and data analyses. All authors revised and approved the final manuscript.

## Supplementary Material

Additional file 1 ***Trypanosoma vivax *** **isolates from South America.** Table comprising all *T. vivax* isolates from Brazil, Venezuela and French Guiana characterized in this study, livestock species and geographic origin, clinical conditions of the infected livestock, and MLG genotypes defined using 7 microsatellite loci. Click here for file

Additional file 2 ***Trypanosoma vivax *** **isolates from Africa.** Table showing all African *T. vivax* isolates, host species and geographic origin, clinical conditions of the infected animals, and microsatellite genotypes (MLGs) defined using 7 microsatellite loci. Click here for file

Additional file 3 **Loci and microsatellite primers employed in the present study.** Primers employed for PCR-amplification, motifs, genes and genome location of microsatellite loci selected for this study. The PCR conditions employed for microsatellite loci amplification are detailed in the Methods Section. Click here for file

## References

[B1] Desquesnes M Livestock trypanosomoses and their vectors in Latin America 2004 OIE, Paris, France: CIRAD-EMVT publication 174 ISBN 92-9044-634-X

[B2] Osório AL Madruga CR Desquesnes M Soares CO Ribeiro LR Costa SC Trypanosoma (Duttonella) vivax: its biology, epidemiology, pathogenesis, and introduction in the new world - a review Mem Inst Oswaldo Cruz 2008 103 1 13 18368231 10.1590/s0074-02762008000100001

[B3] Dayo GK Bengaly Z Messad S Bucheton B Sidibe I Cene B Cuny G Thevenon S Prevalence and incidence of bovine trypanosomosis in an agro-pastoral area of southwestern Burkina Faso Res Vet Sci 2010 88 470 477 20044115 10.1016/j.rvsc.2009.10.010

[B4] Sow A Ganaba R Percoma L Sidibé I Bengaly Z Adam Y Koné P Sawadogo GJ Van Den Abbeele J Marcotty T Delespaux V Baseline survey of animal trypanosomosis in the region of the Boucle du Mouhoun, Burkina Faso Res Vet Sci 2013 94 573 578 23337746 10.1016/j.rvsc.2012.12.011

[B5] Nakayima J Nakao R Alhassan A Hayashida K Namangala B Mahama C Afakye K Sugimoto C Genetic diversity among *Trypanosoma (Duttonella)* vivax strains from Zambia and Ghana, based on cathepsin L-like gene Parasite 2013 20 24 23815966 10.1051/parasite/2013024 PMC3718526

[B6] Majekodunmi AO Fajinmi A Dongkum C Picozzi K Thrusfield MV Welburn SC A longitudinal survey of African animal trypanosomiasis in domestic cattle on the Jos Plateau, Nigeria: prevalence, distribution and risk factors Parasit Vectors 2013 6 239 23958205 10.1186/1756-3305-6-239 PMC3765779

[B7] Biryomumaisho S Rwakishaya EK Melville SE Cailleau A Lubega GW Livestock trypanosomosis in Uganda: parasite heterogeneity and anaemia status of naturally infected cattle, goats and pigs Parasitol Res 2013 112 1443 1450 23344247 10.1007/s00436-013-3275-9

[B8] Sinshaw A Abebe G Desquesnes M Yoni W Biting flies and *Trypanosoma vivax* infection in three highland districts bordering lake Tana, Ethiopia Vet Parasitol 2006 142 35 46 16890359 10.1016/j.vetpar.2006.06.032

[B9] Fikru R Goddeeris BM Delespaux V Moti Y Tadesse A Bekana M Claes F De Deken R Büscher P Widespread occurrence of *Trypanosoma vivax* in bovines of tsetse- as well as non-tsetse-infested regions of Ethiopia: a reason for concern? Vet Parasitol 2012 190 355 361 22858227 10.1016/j.vetpar.2012.07.010

[B10] Salim B Bakheit MA Salih SE Kamau J Nakamura I Nakao R Sugimoto C An outbreak of bovine trypanosomiasis in the Blue Nile State, Sudan Parasit Vectors 2011 4 74 78 21569459 10.1186/1756-3305-4-74 PMC3112103

[B11] Nimpaye H Njiokou F Njine T Njitchouang GR Cuny G Herder S Asonganyi T Simo G *Trypanosoma vivax*, *T. congolense* “forest type” and *T. simiae*: prevalence in domestic animals of sleeping sickness foci of Cameroon Parasite 2011 18 171 179 21678793 10.1051/parasite/2011182171 PMC3671417

[B12] Batista JS Rodrigues CM Olinda RG Silva TM Vale RG Câmara AC Rebouças RE Bezerra FS García HA Teixeira MMG Highly debilitating natural *Trypanosoma vivax* infections in Brazilian calves: epidemiology, pathology, and probable transplacental transmission Parasitol Res 2012 110 73 80 21626156 10.1007/s00436-011-2452-y

[B13] Silva TM Olinda RG Rodrigues CM Câmara AC Lopes FC Coelho WA Ribeiro MF Freitas CI Teixeira MMG Batista JS Pathogenesis of reproductive failure induced by *Trypanosoma vivax* in experimentally infected pregnant ewes Vet Res 2013 44 1 23289625 10.1186/1297-9716-44-1 PMC3598889

[B14] Masake RA The pathogenesis of infection with *Trypanosoma vivax* in goats and cattle Vet Rec 1980 107 551 557 7467108 10.1136/vr.107.24.551

[B15] Magona JW Walubengo J Odimin JT Acute haemorrhagic syndrome of bovine trypanosomosis in Uganda Acta Trop 2008 107 186 191 18599006 10.1016/j.actatropica.2008.05.019

[B16] Moloo SK Orinda GO Sabwa CL Minja SH Masake RA Study on the sequential tsetse-transmitted Trypanosoma congolense, *T. brucei brucei and T. vivax* infections to African buffalo, eland, waterbuck, N’Dama and Boran cattle Vet Parasitol 1999 80 197 213 9950344 10.1016/s0304-4017(98)00209-x

[B17] Rodrigues AC Neves L Garcia HA Viola LB Marcili A Maia da Silva F Sigauque I Batista JS Paiva F Teixeira MMG Phylogenetic analysis of *Trypanosoma vivax* supports the separation of South American/West African from East African isolates and a new *T. vivax*-like genotype infecting a nyala antelope from Mozambique Parasitol 2008 13 1317 1328 10.1017/S0031182008004848 18752705

[B18] Anderson NE Mubanga J Fevre EM Picozzi K Eisler MC Thomas R Welburn SC Characterisation of the wildlife reservoir community for human and animal trypanosomiasis in the Luangwa Valley, Zambia PLoS Negl Trop Dis 2011 5 e1211 21713019 10.1371/journal.pntd.0001211 PMC3119639

[B19] Auty H Anderson NE Picozzi K Lembo T Mubanga J Hoare R Fyumagwa RD Mable B Hamill L Cleaveland S Welburn SC Trypanosome diversity in wildlife species from the serengeti and luangwa valley ecosystems Plos Negl Trop Dis 2012 6 e1828 23094115 10.1371/journal.pntd.0001828 PMC3475651

[B20] Galiza GJ Garcia HA Assis AC Oliveira DM Pimentel LA Dantas AF Simões SV Teixeira MMG Riet-Correa F High mortality and lesions of the central nervous system in Trypanosomosis by *Trypanosoma vivax* in Brazilian hair sheep Vet Parasitol 2011 182 359 363 21664764 10.1016/j.vetpar.2011.05.016

[B21] Da Silva AS Garcia HA Costa MM França RT De Gasperi D Zanette RA Amado JÁ Lopes STA Teixeira MMG Monteiro SG Horses naturally infected by *Trypanosoma vivax* in southern Brazil Parasitol Res 2011 108 23 30 20820805 10.1007/s00436-010-2036-2

[B22] Cadioli FA Barnabé PA Machado RZ Teixeira MC André MR Sampaio PH Fidélis Junior OL Teixeira MMG Marques LC First report of *Trypanosoma vivax* outbreak in dairy cattle in São Paulo state, Brazil Rev Bras Parasitol Vet 2012 21 118 124 22832751 10.1590/s1984-29612012000200009

[B23] Batista JS Rodrigues CMF García HA Bezerra FS Olinda RG Teixeira MMG Soto–Blanco B Association of *Trypanosoma vivax* in extracellular sites with central nervous system lesions and changes in cerebrospinal fluid in experimentally infected goats Vet Res 2011 42 63 69 21569364 10.1186/1297-9716-42-63 PMC3105954

[B24] Kimeto BA Mugera GM Nyaga PN Haemorrhagic pancarditis in cattle infected with *Trypanosoma vivax* Vet Parasitol 1990 34 295 301 2316175 10.1016/0304-4017(90)90076-n

[B25] Guegan F Plazolles N Baltz T Coustou V Erythrophagocytosis of desialylated red blood cells is responsible for anaemia during *Trypanosoma vivax* infection Cell Microbiol 2013 15 1285 1303 23421946 10.1111/cmi.12123

[B26] D’Archivio S Cosson A Medina M Lang T Minoprio P Goyard S Non-invasive in vivo study of the *Trypanosoma vivax* infectious process consolidates the brain commitment in late infections PLoS Negl Trop Dis 2013 7 e1976 23301112 10.1371/journal.pntd.0001976 PMC3536815

[B27] Chamond N Cosson A Blom-Potar MC Jouvion G D'Archivio S Medina M Droin-Bergère S Huerre M Goyard S Minoprio P Trypanosoma vivax infections: pushing ahead with mouse models for the study of Nagana. I. Parasitological, hematological and pathological parameters PLoS Negl Trop Dis 2010 10 e792 20706595 10.1371/journal.pntd.0000792 PMC2919405

[B28] Rodrigues CM Olinda RG Silva TM Vale RG da Silva AE Lima GL Garcia HA Teixeira MMG Batista JS Follicular degeneration in the ovaries of goats experimentally infected with *Trypanosoma vivax* from the Brazilian semi-arid region Vet Parasitol 2013 191 146 153 22921989 10.1016/j.vetpar.2012.08.001

[B29] Batista JS Oliveira AF Rodrigues CM Damasceno CA Oliveira IR Alves HM Paiva ES Brito PD Medeiros JM Rodrigues AC Teixeira MMG Infection by *Trypanosoma vivax* in goats and sheep in the Brazilian semiarid region: from acute disease outbreak to chronic cryptic infection Vet Parasitol 2009 165 131 135 19665308 10.1016/j.vetpar.2009.07.005

[B30] Ventura RM Paiva F Silva RA Takeda GF Buck GA Teixeira MMG *Trypanosoma vivax*: characterization of the spliced-leader gene of a Brazilian stock and species-specific detection by PCR amplification of an intergenic spacer sequence Exp Parasitol 2001 99 37 48 11708832 10.1006/expr.2001.4641

[B31] Paiva F Lemos RAA Nakazato L Brum KB Bernardo KC Madruga CR Schenk MA Trypanosoma vivax em bovinos no Pantanal do Estado do Mato Grosso do Sul, Brasil: II - Inoculação experimental Rev Bras Parasitol Vet 2000 9 135 141

[B32] Garcia HA Garcia ME Perez H Mendoza-Leon A The detection and PCR-based characterization of the parasites causing trypanosomiasis in water-buffalo herds in Venezuela Ann Trop Med Parasitol 2005 99 359 370 15949183 10.1179/136485905X36271

[B33] Garcia HA Garcia ME Pérez G Bethencourt A Zerpa E Pérez H Mendoza–León A Trypanosomiasis in Venezuelan water buffaloes: association of packed-cell volumes with seroprevalence and current trypanosome infection Ann Trop Med Parasitol 2006 100 297 305 16762110 10.1179/136485906X91521

[B34] Cortez AP Ventura RM Rodrigues AC Batista JS Paiva F Añez N Machado RZ Gibson WC Teixeira MMG The taxonomic and phylogenetic relationships of *Trypanosoma vivax* from South America and Africa Parasitol 2006 133 159 169 10.1017/S0031182006000254 16650339

[B35] Cortez AP Rodrigues AC Garcia HA Neves L Batista JS Bengaly Z Paiva F Teixeira MMG Cathepsin L-like genes of *Trypanosoma vivax* from Africa and South America-characterization, relationships and diagnostic implications Mol Cell Probe 2009 23 44 51 10.1016/j.mcp.2008.11.003 19063960

[B36] Dirie MF Murphy NB Gardiner PR DNA fingerprinting of *Trypanosoma vivax* isolates rapidly identifies intraspecific relationships J Euk Microbiol 1993 40 132 134 8461886 10.1111/j.1550-7408.1993.tb04892.x

[B37] Dirie MF Otte MJ Thatthi R Gardiner PR Comparative studies of *Trypanosoma (Duttonella) vivax* isolates from Colombia Parasitol 1993 106 21 29 10.1017/s0031182000074771 8097584

[B38] Mariante AS Egito AA Animal genetic resources in Brazil: result of five centuries of natural selection Theriog 2003 57 223 235 10.1016/s0093-691x(01)00668-9 11775972

[B39] Martínez AM Gama LT Cañón J Ginja C Delgado JV Dunner S Landi V Martín-Burriel I Penedo MC Rodellar C Vega-Pla JL Acosta A Alvarez LA Camacho E Cortés O Marques JR Martínez R Martínez RD Melucci L Martínez-Velázquez G Muñoz JE Postiglioni A Quiroz J Sponenberg P Uffo O Villalobos A Zambrano D Zaragoza P Genetic footprints of Iberian Cattle in America 500 years after the arrival of Columbus PLoS One 2012 7 e49066 23155451 10.1371/journal.pone.0049066 PMC3498335

[B40] Ginja C Penedo MC Melucci L Quiroz J Martínez López OR Revidatti MA Martínez-Martínez A Delgado JV Gama LT Origins and genetic diversity of new world Creole cattle: inferences from mitochondrial and Y chromosome polymorphisms Anim Genet 2010 41 128 141 19817725 10.1111/j.1365-2052.2009.01976.x

[B41] Adams ER Hamilton PB Rodrigues AC Malele II Delespaux V Teixeira MMG Gibson W New *Trypanosoma (Duttonella) vivax* genotypes from tsetse flies in East Africa Parasitol 2010 137 641 650 10.1017/S0031182009991508 19961657

[B42] Adams ER Hamilton PB Gibson WC African trypanosomes: celebrating diversity Trends Parasitol 2010 26 324 328 20382076 10.1016/j.pt.2010.03.003

[B43] Hamilton PB Is *Trypanosoma vivax* genetically diverse? Trends Parasitol 2012 28 173 22459431 10.1016/j.pt.2012.02.003

[B44] Truc P Tiouchichine ML Cuny G Vatunga G Josenando T Simo G Herder S Multiple infections of *Trypanosoma brucei gambiense* in blood and cerebrospinal fluid of human African trypanosomosis patients from Angola: consequences on clinical course and treatment outcome Infect Genet Evol 2012 12 399 402 22285307 10.1016/j.meegid.2012.01.010

[B45] Balmer O Beadell JS Gibson W Caccone A Phylogeography and taxonomy of *Trypanosoma brucei* PLoS Negl Trop Dis 2011 5 e961 21347445 10.1371/journal.pntd.0000961 PMC3035665

[B46] Capewell P Cooper A Duffy CW Tait A Turner CM Gibson W Mehlitz D Macleod A Human and animal trypanosomes in Côte d’Ivoire form a single breeding population PLoS One 2013 8 e67852 23844111 10.1371/journal.pone.0067852 PMC3699513

[B47] Duffy CW Maclean L Sweeney L Cooper A Turner CM Tait A Sternberg J Morrison LJ Macleod A Population genetics of *Trypanosoma brucei rhodesiense*: clonality and diversity within and between foci PLoS Negl Trop Dis 2013 7 e2526 24244771 10.1371/journal.pntd.0002526 PMC3828156

[B48] Morrison LJ Tweedie A Black A Pinchbeck GL Christley RM Schoenefeld A Hertz-Fowler C MacLeod A Turner CM Tait A Discovery of mating in the major African livestock pathogen *Trypanosoma congolense* PLoS One 2009 4 e5564 19440370 10.1371/journal.pone.0005564 PMC2679202

[B49] Simo G Sobgwi PF Njitchouang GR Njiokou F Kuiate JR Cuny G Asonganyi T Identification and genetic characterization of *Trypanosoma congolense* in domestic animals of Fontem in the South-West region of Cameroon Infect Genet Evol 2013 18 66 73 23624186 10.1016/j.meegid.2013.04.019

[B50] Duffy CW Morrison LJ Black A Pinchbeck GL Christley RM Schoenefeld A Tait A Turner CM MacLeod A *Trypanosoma vivax* displays a clonal population structure Int J Parasitol 2009 39 1475 1483 19520081 10.1016/j.ijpara.2009.05.012

[B51] Morlais I Ravel S Grébaut P Dumas V Cuny G New molecular marker for *Trypanosoma (Duttonella) vivax* identification Acta Trop 2001 80 207 213 11700177 10.1016/s0001-706x(01)00160-7

[B52] Biryomumaisho S Katunguka-Rwakishaya E Lubega GW Melville SE Identification of *Trypanosoma vivax* subtypes isolated from cattle and goats using microsatellite markers Vet Arh 2011 81 13 24

[B53] Excoffier L Lischer HE Arlequin suite ver 3.5: a new series of programs to perform population genetics analyses under linux and windows Mol Ecol Resour 2010 10 564 567 21565059 10.1111/j.1755-0998.2010.02847.x

[B54] Peakall R Smouse PE GenAlexX 6: genetic analysis in excel: population genetic software for teaching and research Mol Ecol Notes 2006 6 288 295 10.1093/bioinformatics/bts460 PMC3463245 22820204

[B55] Langella O Populations, 1.2.30: population genetic software (individuals or populations distances, phylogenetic trees) 1999 France: CNRS

[B56] Jin L Chakraborty R Estimation of genetic distance and coefficient of gene diversity from single-probe multilocus DNA fingerprinting data Mol Biol Evol 1993 11 120 127 8121280 10.1093/oxfordjournals.molbev.a040086

[B57] Pritchard JK Stephens M Donnelly P Inference of population structure using multilocus genotype data Genetics 2000 155 945 959 10835412 10.1093/genetics/155.2.945 PMC1461096

[B58] Evanno G Regnaut S Goudet J Detecting the number of clusters of individuals using the software STRUCTURE: a simulation study Mol Ecol 2005 14 2611 2620 15969739 10.1111/j.1365-294X.2005.02553.x

[B59] Fikru R Hagos A Rogé S Reyna-Bello A Gonzatti MI Merga B Goddeeris BM Büscher PA Proline racemase based PCR for identification of *Trypanosoma vivax* in cattle blood PLoS One 2014 9 e84819 24416292 10.1371/journal.pone.0084819 PMC3885604

[B60] Simo G Njitchouang G Melachio TT Njiokou F Cuny G Tazoacha A Population genetics of *Trypanosoma brucei* circulating in *Glossina palpalis palpalis* and domestic animals of the fontem sleeping sickness focus of Cameroon Parasit Vectors 2014 7 156 24690359 10.1186/1756-3305-7-156 PMC4022266

[B61] Jackson AP Berry A Aslett M Allison HC Burton P Vavrova-Anderson J Brown R Browne H Corton N Hauser H Gamble J Gilderthorp R Marcello L McQuillan J Otto TD Quail MA Sanders MJ van Tonder A Ginger ML Field MC Barry JD Hertz-Fowler C Berriman M Antigenic diversity is generated by distinct evolutionary mechanisms in African trypanosome species Proc Natl Acad Sci U S A 2012 109 3416 3421 22331916 10.1073/pnas.1117313109 PMC3295286

[B62] Greif G Ponce de Leon M Lamolle G Rodriguez M Piñeyro D Tavares-Marques LM Reyna-Bello A Robello C Alvarez Valin F Transcriptome analysis of the bloodstream stage from the parasite *Trypanosoma vivax* BMC Genomics 2013 14 149 23497072 10.1186/1471-2164-14-149 PMC4007602

