# Supplementary material for: Microsatellite analysis supports clonal propagation and reduced divergence of Trypanosoma vivax from asymptomatic to fatally infected livestock in South America compared to West Africa
Source: Parasit Vectors. 2014 May 3;7:210. doi: 10.1186/1756-3305-7-210 (PMC4023172; doi:10.1186/1756-3305-7-210)
Supplement: Additional file 1 — Trypanosoma vivax isolates from South America. Table comprising all T. vivax isolates from Brazil, Venezuela and French Guiana characterized in this study, livestock species and geographic origin, clinical conditions of the infected livestock, and MLG genotypes defined using 7 microsatellite loci. [file 1756-3305-7-210-S1.docx]

**Additional file 1.** ***Trypanosoma vivax* isolates from South America:** Table comprising all *T. vivax* isolates from Brazil, Venezuela and French Guiana characterized in this study, livestock species and geographic origin, clinical conditions of the infected livestock, and MLG genotypes defined using 7 microsatellite loci.

| ***T. vivax***  **isolates** | **MLG** | **Host**  **species** | **Geographic**  **origin** | **Date of**  **isolation** | **Livestock infection**  **clinical signs** | **Reference** |
| --- | --- | --- | --- | --- | --- | --- |
| **Brazil** |  |  |  |  |  |  |
| TviBrPA41 | 1 | buffalo | PA | 2008 | asymptomatic ^a^ | this study |
| TviBrPA45 | 1 | buffalo | PA | 2008 | asymptomatic | this study |
| TviBrPA20 | 1 | buffalo | PA | 2009 | asymptomatic | this study |
| TviBrPA24 | 1 | buffalo | PA | 2009 | asymptomatic | this study |
| TviBrPA25 | 1 | buffalo | PA | 2009 | asymptomatic | this study |
| TviBrPA26 | 1 | buffalo | PA | 2009 | asymptomatic | this study |
| TviBrPA28 | 1 | buffalo | PA | 2009 | asymptomatic | this study |
| TviBrPA31 | 1 | buffalo | PA | 2009 | asymptomatic | this study |
| TviBrPA34 | 1 | buffalo | PA | 2009 | asymptomatic | this study |
| TviBrPA36 | 1 | buffalo | PA | 2009 | asymptomatic | this study |
| TviBrPA37 | 1 | buffalo | PA | 2009 | asymptomatic | this study |
| TviBrPA38 | 1 | buffalo | PA | 2009 | asymptomatic | this study |
| TviBrPA39 | 1 | buffalo | PA | 2009 | asymptomatic | this study |
| TviBrPA40 | 1 | buffalo | PA | 2009 | asymptomatic | this study |
| TviBrSP1 | 1 | cattle | SP | 2008 | high parasitemia, low PCV/NS-Fatal | [23] |
| TviBrSP2 | 1 | cattle | SP | 2008 | high parasitemia low PCV/NS-Fatal | [23] |
| TviBrBov1 | 1 | cattle | MS | 2000 | asymptomatic | this study |
| TviBrPB27 | 2 | sheep | PB | 2008 | high parasitemia low PCV/NS-Fatal | [21] |
| TviBrPB28 | 2 | sheep | PB | 2008 | high parasitemia low PCV/NS-Fatal | [21] |
| TviBrPB30 | 2 | sheep | PB | 2008 | high parasitemia low PCV/NS-Fatal | [21] |
| TviBrPB33 | 2 | sheep | PB | 2008 | high parasitemia low PCV/NS-Fatal | [21] |
| TviBrPB36 | 2 | sheep | PB | 2008 | high parasitemia low PCV/NS-Fatal | [21] |
| TviBrPB38 | 2 | sheep | PB | 2008 | high parasitemia low PCV/NS-Fatal | [21] |
| TviBrPB44 | 2 | sheep | PB | 2008 | high parasitemia low PCV/NS-Fatal | [21] |
| TviBrPB45 | 2 | sheep | PB | 2008 | high parasitemia low PCV/NS-Fatal | [21] |
| TviBrPB46 | 2 | sheep | PB | 2008 | high parasitemia low PCV/NS | [21] |
| TviBrPB47 | 2 | sheep | PB | 2008 | high parasitemia low PCV/NS | [21] |
| TviBrPB48 | 2 | sheep | PB | 2008 | high parasitemia low PCV/NS | [21] |
| TviBrPA49 | 2 | cattle | PA | 2008 | asymptomatic | this study |
| TviBrPA50 | 2 | cattle | PA | 2008 | asymptomatic | this study |
| TviBrPA51 | 2 | cattle | PA | 2008 | asymptomatic | this study |
| TviBrRS1 | 3 | horse | RS | 2009 | low parasitemia, low PCV/NS-Fatal | [22] |
| TviBRPA48 | 3 | buffalo | PA | 2009 | asymptomatic | this study |
| TviBrPB50 | 4 | sheep | PB | 2009 | asymptomatic | [21] |
| TviBrPB51 | 5 | buffalo | PB | 2009 | asymptomatic | [21] |
| TviBrPB52 | 5 | sheep | PB | 2009 | asymptomatic | this study |
| TviBrPB49 | 5 | cattle | PB | 2009 | asymptomatic | this study |
| TviBrPA46 | 6 | buffalo | PA | 2009 | asymptomatic | this study |
| TviBrPA47 | 6 | buffalo | PA | 2009 | asymptomatic | this study |
| **Venezuela** |  |  |  |  |  |  |
| TviVzAp1^Ex^ | 7 | cattle | Apu | 2006 | moderate parasitemia low PVC | this study |
| TviVzAp2 | 8 | sheep | Apu | 2006 | moderate parasitemia low PCV | this study |
| TviVzAp5 | 8 | sheep | Apu | 2006 | moderate parasitemia low PCV | this study |
| TviVzGu1 | 8 | buffalo | Gua | 2006 | moderate parasitemia low PCV | this study |
| TviVzAnz1 | 9 | cattle | Anz | 2006 | asymptomatic | this study |
| TviVzAnz3 | 10 | cattle | Anz | 2006 | asymptomatic | this study |
| TviVzCoj^EX^ | 10 | buffalo | Coj | 2006 | asymptomatic | this study |
| French Guiana | |  |  |  |  |  |
| TviGuyane^EX^ | 11 | sheep |  | 1986 | symptomatic | [1] |

MLGs: Multilocus genotypes. Brazilian States: PA, Pará; PB, Paraiba; RS, Rio Grande do Sul; MS, Mato Grosso do Sul, SP, São Paulo. Venezuelan States: Apu, Apure; Anz, Anzoátegui; Gua, Guárico; Coj, Cojedes. EX- expanded by infection of experimental animals; PCV, packed cell volume; NS, neurological signs. a, animals with very low parasitemia detectable only by PCR.
