# Supplementary material for: Microsatellite analysis supports clonal propagation and reduced divergence of Trypanosoma vivax from asymptomatic to fatally infected livestock in South America compared to West Africa
Source: Parasit Vectors. 2014 May 3;7:210. doi: 10.1186/1756-3305-7-210 (PMC4023172; doi:10.1186/1756-3305-7-210)
Supplement: Additional file 2 — Trypanosoma vivax isolates from Africa. Table showing all African T. vivax isolates, host species and geographic origin, clinical conditions of the infected animals, and microsatellite genotypes (MLGs) defined using 7 microsatellite loci. [file 1756-3305-7-210-S2.docx]

**Additional file 2. *Trypanosoma vivax* isolates from Africa**

Table showing all African *Trypanosoma vivax* isolates, host species and geographic origin, clinical conditions of the infected animals, and microsatellite genotypes (MLGs) defined using 7 microsatellite loci.

| ***T. vivax***  **isolates** | **MLG** | **Host**  **species** | **Geographic**  **origin** | **Date**  **of isolation** | **Haematological**  **and clinical signs** | **Reference** |
| --- | --- | --- | --- | --- | --- | --- |
| **West Africa** |  |  |  |  |  |  |
| TviBfL445 | 12 | cow | Burkina Faso | 2008 | parasitemic | this study |
| TviBfMatorkou | 13 | cow | Burkina Faso | 2008 | parasitemic | this study |
| TviBfFolonzo | 14 | cow | Burkina Faso | 2008 | parasitemic | this study |
| TviBfMene | 15 | cow | Burkina Faso | 2008 | parasitemic | this study |
| TviKCA19J56 | 16 | cow | Ghana | 2008 | parasitemic | this study |
| TviDere091 | 17 | cow | Ghana | 2008 | parasitemic | this study |
| TviKC92J28 | 18 | cow | Ghana | 2008 | parasitemic | this study |
| TviKang92 | 19 | cow | Ghana | 2008 | parasitemic | this study |
| TviBan1 | 20 | cow | Benin | 2008 | parasitemic | this study |
| TviBan1.2 | 21 | cow | Benin | 2008 | parasitemic | this study |
| IL700 | 22 | cow | Nigeria | - | - | [41] |
| Gambia ^EX^ | 23 | cow | The Gambia | 2009 | - | [41] |
| **East Africa** |  |  |  |  |  |  |
| TviMzCb12 | 24 | cow | Mozambique | 2007 | low parasitemia | [35] |
| TviMzNy | 25 | nyala | Mozambique | 2006 | high parasitemia | [17] |

MLGs: Multilocus genotyping based on allele combinations from 7 microsatellite loci.

EX- expanded by infection of experimental animals.
