# Supplementary material for: Microsatellite analysis supports clonal propagation and reduced divergence of Trypanosoma vivax from asymptomatic to fatally infected livestock in South America compared to West Africa
Source: Parasit Vectors. 2014 May 3;7:210. doi: 10.1186/1756-3305-7-210 (PMC4023172; doi:10.1186/1756-3305-7-210)
Supplement: Additional file 3 — Loci and microsatellite primers employed in the present study. Primers employed for PCR-amplification, motifs, genes and genome location of microsatellite loci selected for this study. The PCR conditions employed for microsatellite loci amplification are detailed in the Methods Section. [file 1756-3305-7-210-S3.docx]

**Additional file 3: Microsatellite primers and loci employed in the present study**

Primers employed for PCR-amplification, motifs, genes and genome location of microsatellite loci selected for this study. The PCR conditions employed for microsatellite loci amplification are detailed in materials and methods.

| **Primer** | **Sequence (5’- 3’)** | **Motif** | **Gene ^a^** | **Chromosome location^a^** |
| --- | --- | --- | --- | --- |
| **TviMST4F**  **TviMST4R** | GCT CGC CTA AGG TTG TCC GCA TA-6FAM  TTC AAC TGG AGT TGC CAC TGG C | (AC)_18_. ~ 258 pb | hypothetical protein, Glutaredoxin/Thioredoxin-like | Chrom-XI |
| **TviMST7F**  **TviMST7R** | CTT GCC TAG CAT TCC TGA TAC TGA G-6FAM  CAG CAC TGA TTT ACA ATC CCA ATA C | (AG)_21_. ~ 254 pb | Unknown | unknown |
| **TviMST8F**  **TviMST8R** | GAC TTA CAG GTG CGG TAT ACT CAT-6FAM  GAG TCT CGA CTG TCA CAA AGT G | (CT)_19_. ~ 183 pb | conserved hypothetical protein,  Aminotransferase | Chrom-VIII |
| **TviMST10F**  **TviMST10R** | CTA ACT GCG CCA CGT TAA CAG GT-6FAM  CAG GCA AAC AAG GTG CAT ATC G | (CT)_20_. ~ 129 pb | Cytochrome P450 | Chrom-III |
| **TviMST11F**  **TviMST11R** | GCT GCC TTG TAC TGT GAG CCG CTG AT-6FAM  GGA CAG AGT AAG CCA CGT GTA GGT C | (TG)_21_. ~ 180 pb | [Diguanylate phosphodiesterase](http://www.ebi.ac.uk/interpro/IEntry?ac=IPR001633) | Chrom-III |
| **TviMST13F**  **TviMST13R** | TCA ACG ATG GAT AAC ATG TAC C-6FAM  GCC CAG TGG TAG TGT GAG CTA | (AC)_20_. ~ 150 pb | AMN1 Superfamily | Chrom-X |
| **TviMST15F**  **TviMST15R** | TTG TTA CAG CGG CAT TAG TGG C-6FAM  GTG CAG TGC GGC ACA CTA GC | (AC)_20_. ~ 150 pb | [hypothetical protein](http://www.ncbi.nlm.nih.gov/nucleotide/340053916?report=gbwithparts&from=541265&to=541756&RID=UURB4SZV01R) | Chrom-VI |

a, *T. vivax* Y486 ongoing Genome Project from Sanger Institute (http://www.sanger.ac.uk/Projects/T_vivax/).
